# Publisher’s Note

**DOI:** 10.1080/21655979.2024.2326360

**Published:** 2024-03-01

Due to a publisher error, a retraction notice was incorrectly issued on the article ‘Functional analysis of ceRNA network of lncRNA TSIX/miR-34a-5p/RBP2 in acute myocardial infarction based on GEO database’, https://doi.org/10.1080/21655979.2021.2006865. We have now removed the retraction notice.

We apologise to the authors for this error.

